# Ten-Year Experience with Native Joint Septic Arthritis: A Retrospective Cohort Study from a Tertiary Center

**DOI:** 10.3390/jcm14186403

**Published:** 2025-09-10

**Authors:** Pietro Cimatti, Jacopo Ciaffi, Benedetta Dallari, Francesco Amicucci, Giovanni Trisolino, Elisa Storni, Alessandra Maso, Francesco Ursini, Dante Dallari

**Affiliations:** 1 Reconstructive Orthopaedic Surgery and Innovative Techniques-Musculoskeletal Tissue Bank, IRCCS Istituto Ortopedico Rizzoli, via G.C. Pupilli 1, 40136 Bologna, Italy; pietro.cimatti@ior.it (P.C.); benedetta.dallari@ior.it (B.D.); francesco.amicucci@ior.it (F.A.); dante.dallari@ior.it (D.D.); 2 Medicine and Rheumatology Unit, IRCCS Istituto Ortopedico Rizzoli, via G.C. Pupilli 1, 40136 Bologna, Italy; francesco.ursini2@unibo.it; 3 Department of Biomedical and Neuromotor Sciences (DIBINEM), Alma Mater Studiorum University of Bologna, via U. Foscolo 7, 40123 Bologna, Italy; 4 Unit of Pediatric Orthopedics and Traumatology, IRCCS Istituto Ortopedico Rizzoli, via G.C. Pupilli 1, 40136 Bologna, Italy; giovanni.trisolino@ior.it; 5 Laboratory of Microbiology and GMP Quality Control, IRCCS Istituto Ortopedico Rizzoli, via G.C. Pupilli 1, 40136 Bologna, Italy; elisa.storni@ior.it (E.S.); alessandra.maso@ior.it (A.M.)

**Keywords:** septic arthritis, antibiotics, orthopedic surgery, outcomes, predictors

## Abstract

**Background**: Native joint septic arthritis is a severe infection associated with considerable morbidity. The data about the microbiological spectrum, treatment methods, and long-term outcomes are heterogeneous. **Methods**: We performed a decade-long retrospective study encompassing all patients with native joint septic arthritis treated at our institution, a tertiary orthopedic center. Data on demographics, clinical parameters, microbiology, surgical interventions, and antibiotic use were gathered. Outcomes included reoperation, persistent infection and mortality during follow-up. We used logistic regression to identify predictors of adverse outcomes, and Kaplan–Meier analyses to evaluate reoperation-free survival among microbiologic groups. **Results**: A total of 114 patients (103 adults and 11 children) were included. Cultures yielded positive results in 72 out of 103 (70%) adults and 8 out of 11 (73%) children. *Staphylococcus aureus* was the primary pathogen in adults (49% of positives) and children (88%), followed by *coagulase-negative staphylococci*. Antibiotics were administered to all patients, with combinations of at least two molecules in 68% of adults and 91% of children, while surgical intervention predominantly consisted of debridement alone. In adults, an elevated preoperative white blood cell count was associated with unfavorable outcomes in univariate analysis (odds ratio 1.14, 95% confidence interval 1.01–1.30, *p* = 0.040). The Kaplan–Meier analysis revealed no significant differences in reoperation-free survival across microbiologic groups (log-rank *p* = 0.361). **Conclusions**: Over a ten-year period, *Staphylococcus aureus* remained the predominant cause of native joint septic arthritis; however, culture-negative cases and *coagulase-negative staphylococci* were also common. Only preoperative leukocytosis was a predictor of poor outcomes, while microbiologic etiology did not significantly influence the risk of reoperation, potentially indicating early and effective therapy. These findings highlight the intricacy of native joint septic arthritis and the necessity for enhanced diagnostics and prognostic stratification.

## 1. Introduction

Septic arthritis of native joints is a medical emergency that, if not rapidly detected and treated, may result in permanent joint damage, systemic infection, and potentially fatal outcomes [1,2,3]. Although relatively rare, it imposes a significant burden of morbidity and mortality. In adults, long-term functional impairment occurs in up to 40% of cases, with mortality rates varying from 5% to 15%, depending on patient age, comorbidities, and time to intervention [1]. In the pediatric population, septic arthritis may present with subtle or atypical symptoms, and delays in detection or misdiagnosis can lead to growth plate injury and permanent deformities [4].

The estimated incidence of native joint septic arthritis ranges from 4 to 60 cases per 100,000 individuals annually in the general population, but it rises significantly among older adults and patients with risk factors such as rheumatoid arthritis, diabetes mellitus, end-stage renal disease, immunosuppression, recent joint injections, or surgery [5,6,7]. Monoarticular involvement is prevalent, especially in major joints including the knee, hip, and shoulder [8,9]. The clinical manifestation is frequently acute, characterized by joint swelling, erythema, warmth, discomfort, and functional impairment, usually associated with raised inflammatory markers and leukocytosis [8,10]. Nonetheless, unusual or subacute presentations, particularly in immunocompromised patients, can hinder clinical identification [2,3].

*Staphylococcus aureus* (*S. aureus*) is the primary causal agent in adults and children, responsible for 40–60% of culture-positive cases, with methicillin-resistant strains (*MRSA*) more common in healthcare-associated infections and specific geographic regions [11,12]. Streptococci, Gram-negative bacilli, and, in risk populations, anaerobes and fungi may also be implicated [1]. In children under four years, opportunistic organisms like *Kingella kingae* are increasingly identified, especially in Europe and North America, but are often missed by standard culture techniques [13].

Notwithstanding the pivotal role of microbiological confirmation, a significant percentage of cases, particularly in children, remain culture-negative. Retrospective series indicate culture-negative frequencies of 20–30% in adult populations and as high as 60–70% in pediatric cohorts [5,14]. These cases frequently exhibit characteristic clinical and synovial fluid findings, and many respond to empirical treatment [15,16]. The lack of a confirmed pathogen complicates antibiotic choice, restricts pathogen surveillance efforts, and may sometimes indicate misdiagnosed crystal arthropathies or inflammatory arthritis [17]. Innovations in diagnostics, such as the inoculation of synovial fluid into blood culture vials, polymerase chain reaction (PCR) techniques, and next-generation sequencing, have the potential to decrease culture-negativity; nevertheless, they are not yet a common practice in many institutions [18,19].

Despite its clinical significance, septic arthritis is still relatively underexamined in extensive contemporary epidemiological research. A significant portion of the existing studies originates from limited monocentric cohorts, pediatric registries, or administrative databases that lack detailed clinical information [20,21]. Population-level studies, like those from national registries in the United Kingdom and Australia, have provided significant insights into incidence and mortality, but numerous inquiries persist about the changing microbiologic spectrum, diagnostic methodologies, management strategies, and patient outcomes in real-world settings [22,23].

In this regard, retrospective hospital-based cohort studies represent a valuable method to enhance our comprehension of native joint septic arthritis. Major tertiary referral facilities, especially those focused on orthopedics and musculoskeletal medicine, are the ideal setting for identifying a variety of clinical presentations and microbiological patterns, encompassing both prevalent and uncommon etiologies. These environments facilitate the systematic acquisition of demographic, clinical, laboratory, and radiographic data, together with the documentation of microbiological results and patient outcomes.

This study intends to present a thorough, updated analysis of native joint septic arthritis derived from a decade-long retrospective experience at a high-volume orthopedic referral hospital. The aim is to delineate the demographic characteristics, clinical features, microbiologic profiles, and clinical outcomes among an unselected cohort of consecutive adult and pediatric patients.

## 2. Materials and Methods

### 2.1. Study Design and Population

We conducted a retrospective observational study at the IRCCS Istituto Ortopedico Rizzoli, a high-volume tertiary referral hospital specialized in orthopedic surgery and musculoskeletal infections in Italy.

We included all patients diagnosed with primary septic arthritis of a native joint who had surgical intervention during the initial hospital admission at our institution from 1 January 2014, to 31 December 2023. Eligible cases were found by a systematic inquiry of the institutional digital archive (SIR2020), employing a combination of diagnostic and procedural codes. We specifically identified individuals with a discharge diagnosis of septic arthritis and a corresponding procedure code for surgical or arthroscopic debridement of a native joint.

The diagnosis was verified through an exhaustive examination of medical records, encompassing clinical presentation, laboratory data including synovial fluid analysis when available, imaging findings, and surgical documentation. Both monoarticular and oligoarticular manifestations were considered. No age limitations were established and both adult and pediatric patients were included.

Importantly, we enrolled all patients who had a presumptive diagnosis of septic arthritis at the time of admission and treatment. This included individuals with a positive microbiological culture from synovial fluid and/or intraoperative samples, as well as those with negative culture results who were nevertheless treated for septic arthritis based on multidisciplinary clinical assessment. In these culture-negative patients, the diagnosis was corroborated by a convergence of indicative findings, such as purulent synovial fluid with leukocyte counts above 50,000 cells/mm^3^ and/or >90% polymorphonuclear cells, acute inflammation on histological evaluation, consistent clinical manifestations, and the exclusion of a more plausible alternative diagnosis [24].

Patients were excluded if they had a prosthetic joint infection or a secondary septic arthritis resulting from contiguous osteomyelitis or adjacent abscess. Cases with a prior history of surgical interventions on the same joint were also eliminated. Additionally, we excluded patients whose diagnostic evaluation or initial therapy was performed outside our institution, along with those with incomplete or inaccessible clinical records.

In our institution, all patients with a suspicion of septic arthritis are systematically managed with surgical debridement within a short timeframe. As a result, the present cohort corresponds to the total number of patients treated for septic arthritis at our hospital during the study period, with no cases managed exclusively with medical therapy.

### 2.2. Data Collection and Variables

Data were obtained retrospectively from medical records, laboratory databases, surgery reports, and the institutional digital archive. We documented age, sex, affected joint(s), and laterality for each patient. Clinical information encompassed the occurrence of fever, duration of symptoms prior to surgery and comorbidities. Microbiological data covered findings from synovial fluid cultures and intraoperative specimens. Pathogens were categorized according to species identification, specifically noting *S. aureus* and differentiating between methicillin-sensitive (*MSSA*) and methicillin-resistant (*MRSA*) strains, *coagulase-negative staphylococci* (*CoNS*), streptococci, Gram-negative bacilli, and other organisms. In patients with polymicrobial infections, each isolate was counted individually; therefore, the cumulative number of pathogens exceeds the number of culture-positive patients.

In line with institutional protocols, patients with a strong clinical suspicion of septic arthritis typically received immediate surgical debridement without preceding diagnostic arthrocentesis. Consequently, synovial fluid analysis was available only in a limited number of cases. In most patients, microbiological diagnosis depended on intraoperative specimens. We also collected information on previous intra-articular procedures (arthrocentesis or injections) performed within 30 days prior to symptom onset. Baseline laboratory results encompassed peripheral white blood cell (WBC) count, C-reactive protein (CRP), and erythrocyte sedimentation rate (ESR).

The surgical details included the date and type of the initial procedure (such as debridement alone, debridement with resection and spacer, or debridement with arthrodesis), the total number of surgical interventions, and any subsequent surgeries, along with their timing and nature. The antibiotic treatment data encompassed the targeted therapy informed by microbiological results. We did not analyze the initial empirical therapy, which was largely uniform across patients and thus of limited analytical relevance, although it remains important for clinical outcomes.

The evaluated outcomes included mortality during follow-up, persistence or recurrence of infection at follow-up and requirement for additional surgical interventions. The duration of hospital stay, and follow-up period (measured in months) were documented for all patients. Follow-up information was retrospectively retrieved from clinical records of all visits performed at our hospital. In our setting, follow-up visits are usually scheduled at 1 and 3 months after hospital discharge, and then every 6 months for up to 5 years, or earlier if clinically indicated. The interval may be prolonged further if deemed clinically appropriate.

The research was conducted in compliance with the ethical principles of the 1964 Declaration of Helsinki and its later amendments [25]. The protocol was approved by the Area Vasta Emilia Centro Ethics Committee (Comitato Etico AVEC; approval code CE-AVEC 51/2025/Oss/IOR). Informed consent was sought from all patients included in the study; written consent was obtained from those who could be reached, or from their legal guardians in the case of minors. For patients who were deceased or could not be contacted, the requirement for informed consent was waived by the Ethics Committee.

### 2.3. Statistical Analysis

Descriptive statistics were employed to summarize demographic, clinical, laboratory, microbiological, treatment, and outcome variables. Categorical variables were presented as frequencies and percentages, whereas continuous variables were expressed as medians with interquartile ranges (IQR), given the non-normal distribution of most parameters and for consistency across variables. Comparisons between groups were conducted utilizing the Kruskal–Wallis test for continuous variables. Comparisons were performed across four distinct microbiologic groups, categorized based on culture findings from the index surgery: (1) culture-negative cases; (2) *S. aureus*; (3) *CoNS*; (4) other monomicrobial infections. Individuals with polymicrobial infections were excluded from group comparisons.

An exploratory univariate logistic regression analysis was performed on adult patients to identify potential predictors of a composite poor outcome, defined as the occurrence of at least one of the following: death during follow-up, reoperation during follow-up, or persistence of infection at the last available follow-up. The predictor factors comprised age, sex, duration from symptom onset to surgery, length of hospital stay, baseline WBC count, CRP, and microbiologic group. In addition to the univariate analyses, we also performed an exploratory multivariable logistic regression model including age, sex, duration from symptom onset to surgery, baseline WBC count, and microbiologic group (reference: culture-negative cases). Results were reported as odds ratios (ORs) with 95% confidence intervals (CIs).

A time-to-event analysis was conducted on adult patients using unadjusted Kaplan–Meier survival curves to assess the time from the initial surgery to reoperation due to unresolved infection or progression of infection. Patients were categorized according to the four microbiologic groups previously described. Patients who did not undergo reoperation were censored at their most recent follow-up, while those who died without reoperation were censored at the time of death. The log-rank test was employed to evaluate the differences in survival distributions among groups.

In the pediatric subgroup, only descriptive statistics were reported. All statistical analyses were performed using R (version 4.5.1). A two-sided *p* < 0.05 was considered statistically significant.

## 3. Results

### 3.1. Demographic and Baseline Characteristics of Adult Patients

We included 103 adult patients; 69 (67%) were male. Median age was 58.7 years (47.2–70.5). The median time from symptom onset to surgery was 4.0 days (2.0–5.0). Baseline laboratory values were: WBC 9.7 × 10^9^/L (7.5–12.2), CRP 4.1 mg/dL (1.5–9.9), and ESR 80 mm/h (45–110.5). Median hospital stay was 10.0 days (8.0–14.0), and median follow-up was 27.0 months (18.0–39.0). The most frequently affected joints were the knee (53; 52%) and hip (27; 26%), followed by shoulder (14; 14%), elbow (4; 4%), ankle (3; 3%), wrist (1; 1%), and finger/toe (1; 1%). Laterality was right in 62 (60%) and left in 41 (40%). During follow-up, 46 patients (45%) underwent reoperation and 6 (6%) died. The baseline and outcome characteristics of adult patients are summarized in Table 1.

### 3.2. Demographic and Baseline Characteristics of Pediatric Patients

The pediatric cohort comprised 11 patients; 6 (55%) were male. Median age was 5.0 years (3.5–13.0). The median time from symptom onset to surgery was 4.0 days (3.0–5.0). Baseline laboratory values were: WBC 9.1 × 10^9^/L (6.0–11.7), CRP 2.6 mg/dL (0.8–8.8), and ESR 90 mm/h (69–119). Median hospital stay was 10.5 days (9.0–13.0), and median follow-up 30.0 months (14.0–40.0). Joint involvement included knee (4; 36%), hip (3; 27%), shoulder (1; 9%), elbow (1; 9%), finger/toe (1; 9%), and combined hip and knee in 1 (9%); laterality was left in 8 (73%), right in 2 (18%), and bilateral in 1 (9%). During follow-up, 4 (36%) required reoperation and no deaths occurred. The baseline and outcome characteristics of pediatric patients are summarized in Table 2.

### 3.3. Microbiological Characteristics and Treatment of Adult Patients

Cultures were positive in 72/103 cases (70%), including 4 patients with polymicrobial infections. Among positive cultures, *S. aureus* was isolated in 35 cases (49%) (*MSSA* in 28 and *MRSA* in 7), *CoNS* in 20 cases (28%), and other pathogens in 23 cases (32%), including *Pseudomonas aeruginosa* (6), *Enterococcus* spp. (4), *Streptococcus* spp. (3), *Candida* spp. (3), *Serratia marcescens* (1), *Morganella morganii* (1), *Acinetobacter baumannii* (1), *Neisseria* spp. (1), *Enterobacter* spp. (1), *Cutibacterium acnes* (1), and *Citrobacter freundii* (1). Because patients with polymicrobial infections contributed multiple isolates, the cumulative counts of individual pathogens do not equal the total number of culture-positive cases.

Targeted combination antimicrobial therapy was used in 70 patients (68%), and monotherapy in 33 (32%). Frequently employed agents included rifampicin (60; 58%), levofloxacin (31; 30%), minocycline (29; 28%), daptomycin (12; 12%), teicoplanin (9; 9%), trimethoprim/sulfamethoxazole (9; 9%), and ciprofloxacin (8; 8%). In addition, other antibiotics were used in no more than five patients each, including fosfomycin, piperacillin–tazobactam, ertapenem, meropenem, linezolid, cefazolin, oxacillin, and amoxicillin–clavulanate. Three patients with Candida infection received voriconazole. Surgically, debridement only was performed in 76 patients (74%), debridement with resection and spacer in 24 (23%), and debridement with arthrodesis in 3 (3%). The microbiological, therapeutic, and surgical characteristics of adult patients are detailed in Table 1.

### 3.4. Microbiological Characteristics and Treatment of Pediatric Patients

Cultures were positive in 8/11 cases (73%), including 2 polymicrobial infections (25%). Among positive cultures, *MSSA* was isolated in 7 cases (88%) and *CoNS* in 1 case (13%); other organisms included *Pseudomonas aeruginosa*, *Peptostreptococcus* spp., and *Enterobacter* spp. (each in 1 case; 13% among culture-positive). Because patients with polymicrobial infections contributed multiple isolates, the cumulative counts of individual pathogens do not equal the total number of culture-positive cases.

Targeted combination antibiotic therapy was used in 10 patients (91%) and monotherapy in 1 (9%); commonly used agents included rifampicin (8; 73%), levofloxacin (6; 55%), and minocycline (4; 36%). Surgically, debridement only was performed in 10 patients (91%) and debridement with arthrodesis in 1 patient (9%). The microbiological, therapeutic, and surgical characteristics of pediatric patients are detailed in Table 2.

### 3.5. Inflammatory Markers, Clinical Parameters, and Hospital Stay According to Microbiologic Group in Adult Patients

**Figure 1:**
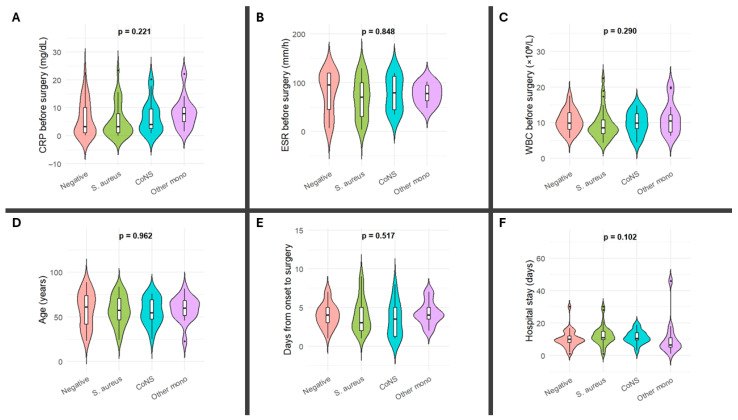
Violin plots comparing baseline clinical and laboratory parameters across microbiological groups in adult patients with native joint septic arthritis. Panels show (**A**) C-reactive protein (CRP) before surgery, (**B**) erythrocyte sedimentation rate (ESR) before surgery, (**C**) white blood cell count (WBC) before surgery, (**D**) age, (**E**) days from symptom onset to surgery, and (**F**) length of hospital stay. Culture-negative cases are shown in light red, *Staphylococcus aureus* in green, *coagulase-negative staphylococci* (*CoNS*) in light blue, and other monomicrobial infections in light purple. *p*-values correspond to Kruskal–Wallis tests comparing medians across groups.

Among adult patients, median age was similar across microbiologic groups, ranging from 54.2 years (IQR 42.5–70.2) in the *CoNS* group to 60.9 years (IQR 41.7–73.7) in the culture-negative group (*p* = 0.962). The median time from symptom onset to surgery was shortest in the *S. aureus* group (3.0 days, IQR 2.0–5.5) and longest in the culture-negative group (4.0 days, IQR 3.0–5.0), with no statistically significant difference between groups (*p* = 0.517). Baseline WBC count before surgery showed no significant variation (*p* = 0.290), with median values ranging from 8.5 × 10^9^/L (IQR 6.9–10.9) in *S. aureus* infections to 10.4 × 10^9^/L (IQR 7.3–13.9) in other monomicrobial infections. Median CRP values were highest in the “other monomicrobial infections” group (7.9 mg/dL, IQR 5.1–12.4) and lowest in the *S. aureus* group (3.2 mg/dL, IQR 1.0–9.0), but differences were not significant (*p* = 0.221). ESR levels were elevated in all groups, with median values ranging from 70 mm/h (IQR 30–112) in *S. aureus* cases to 95 mm/h (IQR 46–120) in culture-negative cases (*p* = 0.848). Median hospital stay was shortest in other monomicrobial infections (6.5 days, IQR 5.0–8.3) and longest in *S. aureus* infections (11.0 days, IQR 9.8–13.5), although this trend did not reach statistical significance (*p* = 0.102). Baseline laboratory and clinical characteristics across microbiological groups are illustrated in the violin plots (Figure 1).

### 3.6. Logistic Regression and Reoperation-Free Survival Analysis

In the univariate logistic regression analysis, higher preoperative WBC count was the only variable significantly associated with the composite poor outcome (OR = 1.14, 95% CI 1.01–1.30; *p* = 0.040). Age (per year; OR = 1.01, 95% CI 0.99–1.04; *p* = 0.374), female sex versus male (OR = 1.49, 95% CI 0.63–3.61; *p* = 0.364), days from symptom onset to surgery (per day; OR = 1.00, 95% CI 0.81–1.23; *p* = 0.977), length of hospital stay (per day; OR = 0.98, 95% CI 0.92–1.04; *p* = 0.538), and preoperative CRP levels (per mg/dL; OR = 0.97, 95% CI 0.91–1.04; *p* = 0.448) were not significantly associated with the outcome. Compared with culture-negative cases, *S. aureus* infection (OR = 0.67, 95% CI 0.25–1.77; *p* = 0.421), *CoNS* infection (OR = 0.51, 95% CI 0.15–1.63; *p* = 0.256), and other monomicrobial infections (OR = 0.95, 95% CI 0.27–3.46; *p* = 0.933) were not significantly associated with the outcome.

However, in the exploratory multivariable model including age, sex, days from symptom onset to surgery, baseline WBC count, and microbiologic group, all associations lost statistical significance. In particular, the association of preoperative WBC count with adverse outcomes, which was significant in the univariate analysis, became non-significant although borderline (*p* = 0.051).

**Figure 2:**
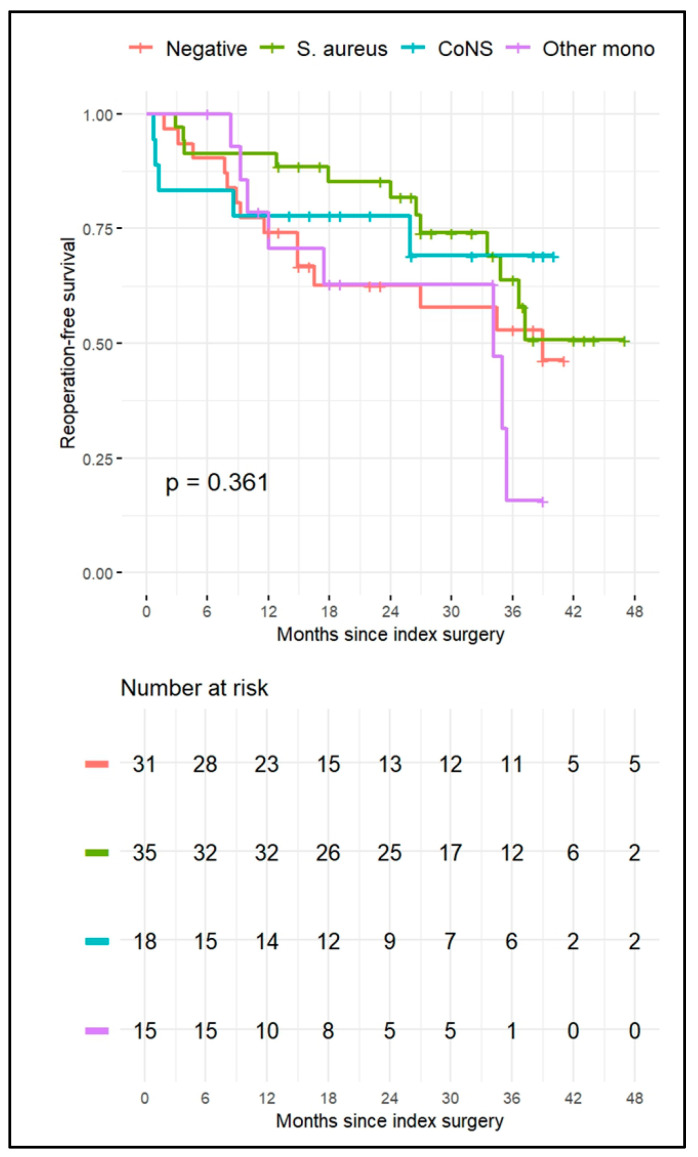
Kaplan–Meier survival curves showing event-free survival (defined as absence of reoperation or progression of infection) stratified by microbiological group in adult patients with native joint septic arthritis. Groups include culture-negative (light red), *Staphylococcus aureus* (green), coagulase-negative staphylococci (*CoNS*, light blue), and other monomicrobial infections (light purple). Numbers at risk are reported at 0, 6, 12, 24, 36, and 48 months. The log-rank test did not reveal statistically significant differences between groups (*p* = 0.361).

Kaplan–Meier survival analysis showed no statistically significant difference in event-free survival between microbiologic groups (log-rank *p* = 0.361). At 12 months, estimated survival probabilities were 91% (95% CI 83–100%) for *S. aureus* infections, 78% (95% CI 61–100%) for *CoNS*, 74% (95% CI 60–91%) for culture-negative cases, and 71% (95% CI 50–100%) for other monomicrobial infections. At 24 months, corresponding survival rates were 82%, 78%, 63%, and 63%, respectively, while at 48 months they declined to 51%, 69%, 46%, and 16%. The survival curves are illustrated in Figure 2.

## 4. Discussion

This retrospective cohort study offers a thorough ten-year analysis of native joint septic arthritis cases treated at a high-volume tertiary orthopedic referral facility, including both adult and pediatric patients. By synthesizing microbiological, clinical, and outcome data, we sought to define the epidemiological spectrum of this condition and investigate potential predictive factors for poor outcomes.

In our cohort, cultures of surgical specimens tested positive in the majority of cases with *S. aureus*—primarily *MSSA*—being the prevalent pathogen in both adults and children. *CoNS* ranked as the second most frequent isolate in adults, although other monomicrobial pathogens were also detected, including Gram-negative bacilli and fungi. Our pediatric findings align with existing research indicating that *S. aureus*—primarily *MSSA*—continues to be the predominant causative agent, whereas *Kingella kingae* and Gram-negative bacteria are rare infections [26]. Culture-negative cases accounted for 30% of adults and 27% of children, consistent with previously reported percentages for adult populations but inferior to other pediatric studies, where figures up to 60–70% had been described [4,23,27,28,29]. At our institution, only conventional culture-based techniques are employed for pathogen identification, which may partly explain the proportion of culture-negative cases observed. This highlights the relevance of optimizing microbiological sampling, including the inoculation of synovial fluid into blood culture bottles and molecular diagnostics such as PCR or next-generation sequencing, which may improve pathogen detection but remain underutilized [17,18,19,30].

Most patients received combination antimicrobial therapy, frequently using rifampicin-based regimens. Debridement was the primary surgical method, with more extensive treatments used mostly in adults. Additionally, in adults an elevated preoperative WBC count was identified as the only significant predictor of the composite poor outcome, while Kaplan–Meier analysis revealed no statistically significant differences in reoperation-free survival among microbiologic groups.

The correlation between elevated preoperative WBC count and unfavorable outcomes in the univariate analysis suggests that the systemic inflammatory burden at presentation could reflect a more active or advanced infection. This corresponds with previous research indicating leukocytosis as a predictor of complex outcomes, such as the necessity for reoperation and prolonged hospitalization [31,32,33]. However, in our exploratory multivariable analysis this association did not retain statistical significance, likely reflecting the limited sample size and statistical power of our study. Notably, CRP levels, the duration from symptom onset to surgery, and microbiologic etiology did not significantly affect outcomes in our adult population.

The finding of no correlation between *S. aureus* infection and poorer prognosis contrasts with previous work, which has associated *S. aureus*—especially *MRSA*—with more severe disease and elevated failure rates [34]. Previous research identified *S. aureus* as a predictor of single debridement failure, while methicillin resistance independently elevates the probability of treatment failure in native joint septic arthritis [34,35]. This divergence might imply an effect of early surgical intervention and rigorous care measures employed at our center on the reduction in pathogen-specific risks. Evidence suggests that coordinated, prompt surgical intervention combined with targeted treatment protocols can yield superior outcomes and permit shorter antibiotic courses following accurate lavage in selected joints [36,37].

The Kaplan–Meier curves showed no statistically significant differences in reoperation-free survival among microbiologic groups, despite descriptive patterns were observed. Infections caused by *S. aureus* had elevated early survival rates relative to other groups, although this benefit waned over time. The “other monomicrobial” category, encompassing many challenging infections, exhibited the most significant reduction after 48 months. Nonetheless, the limited group sizes and the exclusion of polymicrobial cases from the survival analysis hinder clear conclusions.

In this regard, our hospital benefits from a dedicated antimicrobial stewardship program and a specialized multidisciplinary team managing osteoarticular infections, encompassing native septic arthritis, prosthetic joint infections, and musculoskeletal soft-tissue infections [38]. It is therefore plausible that the absence of substantial differences between *S. aureus* and other microbiological groups in our cohort may reflect this structured expertise, as well as the rapidity of both surgical and clinical–antibiotic interventions. However, we acknowledge that the infection spectrum and management strategies observed in such a highly specialized surgical setting may not be entirely generalizable to other institutions, where different diagnostic and therapeutic approaches are adopted.

Our findings highlight numerous important considerations for clinical practice. Although *S. aureus* is the predominant pathogen, clinicians should stay vigilant for *CoNS* and several less frequent pathogens. Secondly, culture-negative cases persist after surgical sampling, highlighting the necessity for enhanced diagnostic approaches. The finding that only preoperative WBC count was associated with adverse outcomes in the univariate analysis underscores the necessity for early detection and vigorous intervention in individuals with significant systemic inflammation.

A major strength of the present research is the extensive, decade-long dataset from a tertiary referral facility, including full clinical, microbiological, and outcome data for both adult and pediatric native joint septic arthritis. The standardized surgical and antimicrobial treatments employed throughout the study period improve the internal consistency of the results. The incorporation of both culture-positive and culture-negative instances provides a comprehensive perspective of actual clinical practice.

However, limitations must be acknowledged. The retrospective design implies risks of incomplete data and unmeasured confounding. The monocentric framework, although facilitating complete case documentation, may restrict generalizability to other healthcare systems characterized by diverse patient demographics or management protocols. The sample size, especially in pediatric and particular microbiologic subgroups, limits statistical power for identifying differences in outcomes. The exclusion of polymicrobial cases from comparative analyses, while methodologically essential, precludes the interpretation of survival outcomes for this patient subset, which may exhibit more intricate clinical trajectories. Finally, although we performed an exploratory multivariable analysis, no independent predictors of adverse outcomes could be confirmed, and the limited sample size still prevents conclusive interpretations.

Future multicenter prospective studies are essential to corroborate our findings and to more accurately delineate prognostic variables, especially for emerging pathogens and antibiotic resistance profiles. The incorporation of advanced microbiological techniques may diminish culture-negativity and facilitate more personalized treatment. Longitudinal evaluation of functional outcomes, especially in pediatric patients, is necessary to fully understand the impact of native joint septic arthritis beyond infection management. Finally, research examining the efficacy of early, targeted combination antibiotic therapy in diminishing recurrence rates would be useful.

## 5. Conclusions

In conclusion, this decade-long retrospective analysis of native joint septic arthritis cases at a tertiary orthopedic center confirms *S. aureus* as the primary pathogen in both adults and children, with *CoNS* and various other organisms also playing substantial roles. Culture-negative cases remain frequent. Of the variables examined, only an elevated preoperative WBC count was a predictor of adverse outcomes in the univariate analysis, while the microbiologic group did not significantly affect the risk of undergoing reoperation. These findings highlight the intricacies of managing native joint septic arthritis and the persistent requirement for improved diagnostics and prognostic stratification to guide treatment.

## Figures and Tables

**Table 1:** Clinical, microbiological, therapeutic, and surgical characteristics of adult patients with native joint septic arthritis.

Adult Patients	N = 103
Males, n (%)	69 (67)
Age (years), median (IQR)	58.7 (47.2–70.5)
Days from symptom onset to surgery, median (IQR)	4.0 (2.0–5.0)
**Site**	
Knee, n (%)	53 (52)
Hip, n (%)	27 (26)
Shoulder, n (%)	14 (14)
Elbow, n (%)	4 (4)
Ankle, n (%)	3 (3)
Wrist, n (%)	1 (1)
Finger/Toe, n (%)	1 (1)
**Laterality**	
Right side, n (%)	62 (60)
Left side, n (%)	41 (40)
**Baseline laboratory tests**	
WBC (×10^9^/L), median (IQR)	9.7 (7.5–12.2)
CRP (mg/dL), median (IQR)	4.1 (1.5–9.9)
ESR (mm/h), median (IQR)	80 (45–111)
**Medical history**	
Autoimmune rheumatic or inflammatory disease, n (%)	17 (17)
Diabetes, n (%)	17 (17)
Insulin therapy, n (%)	5 (5)
Oral antidiabetic therapy only, n (%)	9 (9)
Oral therapy + glucagon-like peptide-1 receptor agonist, n (%)	3 (3)
History of cancer, n (%)	13 (13)
HIV positivity, n (%)	3 (3)
History of drug abuse, n (%)	9 (9)
History of alcohol abuse, n (%)	10 (10)
Prior intra-articular procedures (<30 days), n (%)	9 (9)
Ongoing treatment with glucocorticoids, n (%)	9 (9)
Isolated pathogens *	
Culture positive, n (%)	72 (70)
Polymicrobial infection, n (%)	4 (6)
*MSSA*, *n* (%)	28 (39)
*MRSA*, *n* (%)	7 (10)
*CoNS*, *n* (%)	20 (28)
*Pseudomonas aeruginosa*, *n* (%)	6 (8)
*Enterococcus* spp., *n* (%)	4 (6)
*Streptococcus* spp., *n* (%)	3 (4)
*Candida* spp., *n* (%)	3 (4)
*Serratia marcescens*, *n* (%)	1 (1)
*Morganella morganii*, *n* (%)	1 (1)
*Acinetobacter baumannii*, *n* (%)	1 (1)
*Neisseria* spp., *n* (%)	1 (1)
*Enterobacter* spp., *n* (%)	1 (1)
*Cutibacterium acnes*, *n* (%)	1 (1)
*Citrobacter freundii*, *n* (%)	1 (1)
**Surgical treatment**	
Debridement only, n (%)	76 (74)
Debridement + resection + spacer, n (%)	24 (23)
Debridement + arthrodesis, n (%)	3 (3)
Most frequent targeted antimicrobial treatment **	
Combination antibiotic therapy, n (%)	70 (68)
Antibiotic monotherapy, n (%)	33 (32)
Rifampicin, n (%)	60 (58)
Levofloxacin, n (%)	31 (30)
Minocycline, n (%)	29 (28)
Daptomycin, n (%)	12 (12)
Teicoplanin, n (%)	9 (9)
Trimethoprim/sulfamethoxazole, n (%)	9 (9)
Ciprofloxacin, n (%)	8 (8)
Hospital stay, days, median (IQR)	10.0 (8.0–14.0)
Follow-up duration, months, median (IQR)	27.0 (18.0–39.0)
Reoperation, n (%)	46 (45)
Death during follow-up, n (%)	6 (6)

Abbreviations: *CoNS*, *coagulase-negative staphylococci*; CRP, C-reactive protein; ESR, erythrocyte sedimentation rate; IQR, interquartile range; spp., species; WBC, white blood cell. * Patients with polymicrobial infections contributed more than one isolate; therefore, the sum of pathogens reported exceeds the total number of culture-positive cases. ** Only antimicrobial agents used in more than 5 patients are reported.

**Table 2:** Clinical, microbiological, therapeutic, and surgical characteristics of pediatric patients with native joint septic arthritis.

Pediatric Patients	N = 11
Males, n (%)	6 (55)
Age (years), median (IQR)	5 (3.5–13)
Days from symptom onset to surgery, median (IQR)	4.0 (3.0–5.0)
**Site**	
Knee, n (%)	4 (36)
Hip, n (%)	3 (27)
Shoulder, n (%)	1 (9)
Elbow, n (%)	1 (9)
Finger/Toe, n (%)	1 (9)
Hip and knee, n (%)	1 (9)
**Laterality**	
Right side, n (%)	2 (18)
Left side, n (%)	8 (73)
Bilateral, n (%)	1 (9)
**Baseline laboratory tests**	
WBC (×10^9^/L), median (IQR)	9.1 (6.0–11.7)
CRP (mg/dL), median (IQR)	2.6 (0.8–8.8)
ESR (mm/h), median (IQR)	90 (69–119)
**Medical history**	
Autoimmune rheumatic or inflammatory disease, n (%)	1 (9)
History of cancer, n (%)	1 (9)
Isolated pathogens *	
Culture-positive, n (%)	8 (73)
Polymicrobial infection, n (%)	2 (25)
*MSSA*, *n* (%)	7 (88)
*CoNS*, *n* (%)	1 (13)
*Pseudomonas aeruginosa*, *n* (%)	1 (13)
*Peptostreptococcus* spp., *n* (%)	1 (13)
*Enterobacter* spp., *n* (%)	1 (13)
**Surgical treatment**	
Debridement only, n (%)	10 (91)
Debridement + arthrodesis, n (%)	1 (9)
**Most frequent targeted antimicrobial treatment**	
Combination antibiotic therapy, n (%)	10 (91)
Antibiotic monotherapy, n (%)	1 (9)
Rifampicin, n (%)	8 (73)
Levofloxacin, n (%)	6 (55)
Minocycline, n (%)	4 (36)
Hospital stay, days, median (IQR)	10.5 (9.0–13.0)
Follow-up duration, months, median (IQR)	30.0 (14.0–40.0)
Reoperation, n (%)	4 (36)
Death during follow-up, n (%)	0

Abbreviations: *CoNS*, *coagulase-negative staphylococci*; CRP, C-reactive protein; ESR, erythrocyte sedimentation rate; IQR, interquartile range; spp., species; WBC, white blood cell. * Patients with polymicrobial infections contributed more than one isolate; therefore, the sum of pathogens reported exceeds the total number of culture-positive cases.

## Data Availability

The data that support the findings of this study are institutional and contain sensitive patient information. They are not publicly available due to regulatory and ethical constraints. Data may be made available on reasonable request and pending approval by the appropriate institutional authorities.
